# Frequency and Distribution of Lymphatic Filariasis in Somalia: A Single‐Center Experience

**DOI:** 10.1155/jotm/7385823

**Published:** 2026-05-15

**Authors:** Ahmed Mohamed Ali, Ahmet Dogan, Fardowso Ali Mohamud, Tigad Abdisad Ali, Suad Abdikarim Isse, Abdijalil Abdullahi Ali

**Affiliations:** ^1^ Department of Infectious Diseases and Clinical Microbiology, Turkey Recep Tayyip Erdoğan Training and Research Hospital, Mogadishu, Somalia; ^2^ Department of Infectious Diseases and Clinical Microbiology, Bolu Abant İzzet Baysal University İzzet Baysal Training and Research Hospital, Bolu, Turkey; ^3^ Intern Doctor at Zamzam University of Science and Technology, Mogadishu, Somalia; ^4^ Department of İnfection Prevention and Control in Mogadishu, Turkey Recep Tayyip Erdoğan Training and Research Hospital, Mogadishu, Somalia; ^5^ Department of Cardiovascular Surgery in Mogadishu, Turkey Recep Tayyip Erdoğan Training and Research Hospital, Mogadishu, Somalia

**Keywords:** filariasis, frequency, lymphatic, Somalia

## Abstract

“Filariasis is a chronic, mosquito‐borne parasitic infection. Chronic infection can lead to swelling of extremities, hydroceles, and testicular masses.”

**Purpose:**

The study aimed to evaluate the frequency and distribution of filariasis in Somalia.

**Methods:**

Patients were assessed using clinical examination, cytology, peripheral blood smears, and Doppler ultrasonography. Data were analyzed with IBM SPSS V23. Results were presented as tables, graphs, and frequencies (%).

**Result:**

A total of 22 cases of lymphatic filariasis were assessed in our hospital’s infectious diseases department during follow‐up from March 2018 to December 2023. When the regions of residence of the cases were questioned, it was found that a high proportion of the cases were presented from the same region (Mog‐86%). To a lesser extent, there were applicants from different regions. When the occupations of the cases were questioned, the majority were retired or housewives. When the presenting complaints and physical examination findings were analyzed, it was observed that the right or left extremity, or both, was usually affected. Swelling was the predominant presenting symptom. Genital involvement was detected in about 41% of cases. More than half of them had a history of mosquito bites.

**Conclusion:**

Given LF’s effects on public health, it remains an important public health problem in Somalia.

## 1. Introduction

Lymphatic filariasis (LF) is a tropical, often overlooked parasitic disease, most commonly caused by *Wuchereria bancrofti*. The distribution of these parasites varies, but transmission is more likely in areas with high mosquito density. LF is found in more than 70 countries worldwide, especially in Africa, Brazil, the Dominican Republic, Guyana, and Haiti [1], with an estimated 120 million people infected [2]. *Mansonella perstans* is the most common isolate in Africa but may be confused with *Loa loa* in blood smears during diagnosis [3]. Many cases may be asymptomatic, but long‐term symptoms can include lymphedema, leg swelling, hydrocele or genital swelling in men, hardening or thickening of the skin, and persistent coughing, wheezing, or shortness of breath [4]. Microscopic analysis of blood or antibody detection is a common diagnostic method. Histopathological sampling may also be helpful [5]. Synthetic drugs such as ivermectin, doxycycline, albendazole, and suramin are among the treatment options [6]. The World Health Organization (WHO) recommends a triple‐drug combination (ivermectin, diethylcarbamazine, and albendazole) for eradication [7]. The Global Program for the Elimination of LF (GPELF) aims to eradicate LF by 2030. However, there remains a lack of enriched literature on LF management and disease prevalence [8]. Despite global elimination efforts, significant gaps persist in real‐world clinical data on its epidemiology and clinical characteristics in resource‐limited settings such as Somalia. Therefore, this study aimed to evaluate the demographic characteristics, clinical presentations, and geographical distribution of patients diagnosed with LF in a tertiary care center in Somalia.

## 2. Methods

This study included all cases of LF diagnosed at our center between March 2018 and December 2023. Given the retrospective, descriptive design, no formal sample size calculation was performed, and all eligible patients within the study period were included. Twenty‐two patients diagnosed with infectious diseases were included in the infectious disease department’s follow‐up program. Patients were assessed using clinical evaluation, cytology, peripheral blood smears, and Doppler ultrasonography (DUSG).

## 3. Statistical Analysis

Data analysis was performed using SPSS Version 23. Continuous variables were expressed as median (±standard deviation). Categorical variables were expressed as frequency and percentage. Results were presented as tables and graphs.

## 4. Results

Twenty‐two cases were included. Males made up 54.5%. The mean age was 50.5 ± 20.8 years. Most were outpatients with no comorbid disease. Diagnoses were primarily based on DUSG and clinical findings. Twenty‐seven percent had a two‐week treatment history; one underwent surgery (Table 1).

**TABLE 1:** Some parameters of the cases.

Parameters		*N*	%
Gender	Male	12	54.5
	Female	10	45.5

Age (*M* + SD)	50.50 ± 20.8 (16–74)		

Follow‐up	Outpatient	21	95.5
	Hospitalized	1	4.5

Comorbidity	DM	1	4.5
	HT	2	9.1
	HT and DM	1	4.5
	No	18	81.8

Diagnostic method	Clinically	2	9.1
	Clinically and DUSG	4	18.2
	Cytology and clinically	2	9.1
	Cytology and DUSG	1	4.5
	DUSG	7	31.8
	Microbiology and cytology	1	4.5
	Microbiology smear and clinically	1	4.5
	Microbiology smear and cytology	3	13.6
	Microbiology smear and DUSG	1	4.5

Duration of treatment	1 Month	3	13.6
	1 Week	1	4.5
	2 Months	3	13.6
	2 Weeks	6	27.3
	3 Months	1	4.5
	3 Weeks	2	9.1
	6 Months	2	9.1
	No treatment	4	18.2

Surgery	No	21	95.5
	Yes	1	4.5

*Note:* DUSG, Doppler ultrasonography; HT, hypertension; M, median.

Abbreviations: DM, diabetes mellitus; SD, standard deviation.

Most cases showed right, left, or both extremities affected. Swelling was most common. About 41% had genital involvement. Over half reported mosquito bites (Table 2).

**TABLE 2:** Complaints and physical examination findings of the cases.

Complaints and physical examination findings		*N*	%
Complaint	Bilateral lower limb edema	1	4.5
	Left leg swelling	6	27.3
	Leg pain, swelling	3	13.6
	Leg pain, swelling and fever	1	4.5
	Leg swelling	1	4.5
	Right leg swelling	2	9.1
	Right leg swelling	6	27.3
	Right leg swelling and pain	1	4.5
	Right‐leg swelling and scrotal edema	1	4.5

Genital involvement	No	13	59.1
	Yes	9	40.9

Extremity involvement	Bilateral	1	4.5
	Left leg	6	27.3
	Lower lımb	5	22.7
	Right leg	10	45.5

Mosquito bite	No	10	45.5
	Yes	12	54.5

**FIGURE 1:**
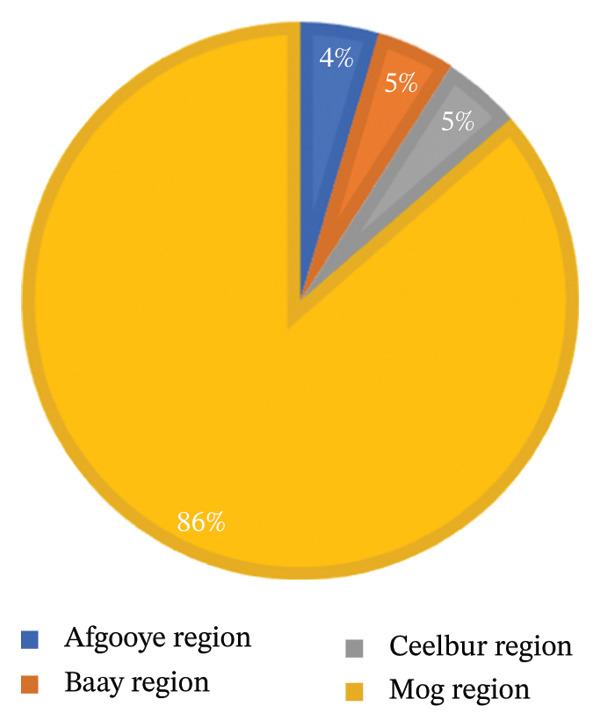
Regions where cases live.

A high proportion of cases were from the same region (the Mog region‐86%), with fewer applicants from other regions (Figure 1).

**FIGURE 2:**
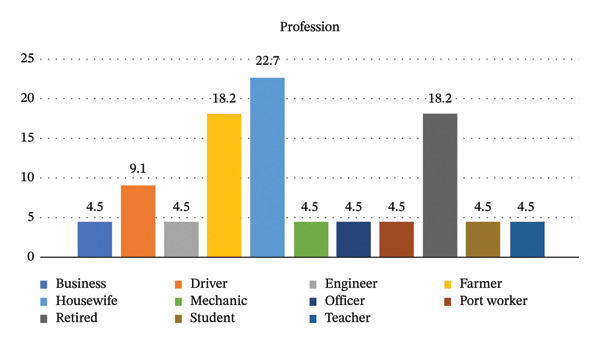
Occupations (%).

Most cases were retired or housewives (Figure 2).

During the physical examination, photographs of the lesion site and microbiologic smear samples were recorded. One example is shown in Figure 3.

FIGURE 3(a) Appearance of filariasis with left extremity involvement. (b) Microbiologic smear of microfilariae of *Wuchereria bancrofti*.

**Figure:**
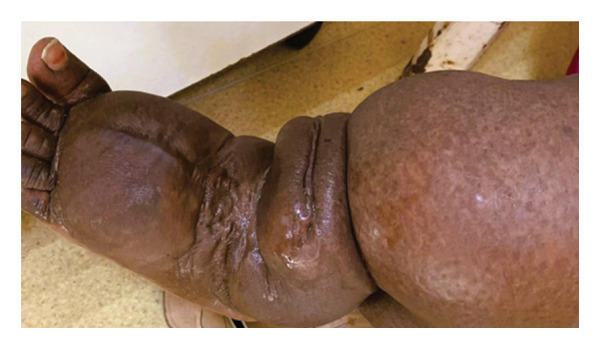
(a)

**Figure:**
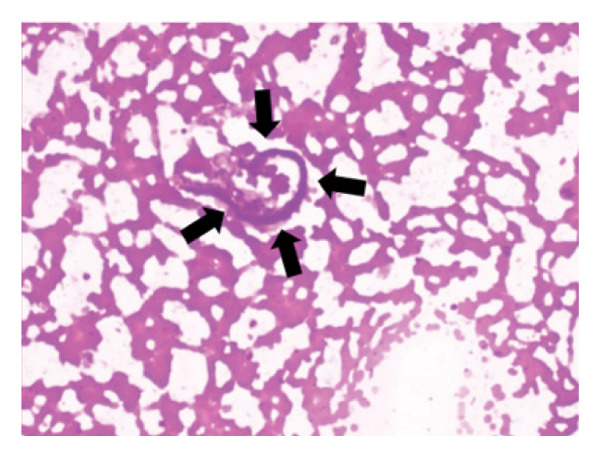
(b)

## 5. Discussion

In this study, we evaluated the demographic characteristics, clinical findings, and geographical distribution of LF cases in Somalia. Our findings demonstrated that the disease predominantly affects individuals from specific endemic regions, with lower‐extremity involvement and swelling as the most common clinical presentation. In addition, a considerable proportion of patients presented with genital involvement. Especially in developing countries in Africa, it is inevitable to encounter difficulties in LF management. Difficulties in diagnosis, access to treatment, and receipt of health services are examples of these difficulties [9–11]. Microbiology smear and clinically, cytology, and DUSG are the methods that can be used in our center. Serological tests, immunofluorescence, or a molecular‐based approach are not possible. Since it is difficult to isolate the agent with microbiological smears, clinical and DUSG have generally been the most helpful methods.

Different clinical involvements can be seen in cases. One of the common clinical involvements is the urogenital system [12]. Genital involvement was observed in almost half of our cases. Lower extremities are usually affected. In particular, the right lower extremity was the most affected organ. However, the head and neck regions may be affected less frequently [13]. Clinical examinations are a very important step in diagnosis. Especially, swelling is a common symptom. Pain and edema may occasionally accompany the disease. Fever, chills, eosinophilia, granulomatous lesions, lymphangitis, lymphadenitis, epididymal orchitis, and lymphadenopathy may also be present [14].

One of the most important challenges for physicians in managing filariasis is the treatment and eradication of the disease. In case‐based approaches, treatment periods can be very long due to partial response to treatments. Sometimes surgical treatment may be necessary. In our cases, patients who needed treatment for up to 6 months were identified. Only one of our cases required surgery. Nowadays, studies on the triple antibiotic combination for the treatment of filariasis have shown its effectiveness [15, 16]. However, the lack of literature on the triple‐combination response across all types of disease‐causing pathogens remains a problem [17].

Occupational groups among the cases are another factor that affects the disease’s incidence. The female gender is at a higher risk. However, the probability of clinical pathology among housewives is, interestingly, higher than in other occupational groups.

The age factor is considerably lower in transmission. Given the high mosquito exposure in Africa, it is now clear that transmission occurs from childhood. However, multiple exposures are necessary for the clinic to occur, unlike malaria [18, 19].

Living locations are another important factor in transmission [20, 21]. Clinical pathology is more likely to occur, especially in rural areas with poor populations. Inadequate mosquito spraying and deficiencies in other prevention methods may affect this. In Somalia, disease management is limited, with no government or international support for neglected tropical diseases. In addition, the subsequent civil war wreaked ruin on Somalia’s public healthcare system. The last 3 decades of armed conflicts, lack of functioning government, economic collapse, and disintegration of the health system and other public services, together with recurrent droughts and famines, have turned Somalia into one of the world’s most difficult environments for survival. Even though the LF‐endemic population is from South Somalia, they lack government health aid for LF and have no support from international partners, which has caused them to regress in providing care for LF and associated complications. To manage their condition and prevent its growth, people who have lymphedema must have access to continuous therapy throughout their entire life. Considering the effects of LF on public health, it is still considered to be an important health problem for people from Somalia.

### 5.1. Study Limitations

This study has several limitations. First, the relatively small sample size and single‐center design limit the generalizability of the findings. Second, due to limited laboratory infrastructure, advanced diagnostic methods, such as molecular assays and serological tests, could not be performed, potentially leading to underdiagnosis or misclassification. Third, the retrospective design may have resulted in incomplete clinical data and potential selection bias. Despite these limitations, the study provides valuable real‐world data from a region with scarce epidemiological data.

## 6. Conclusion

LF remains a significant public health concern in Somalia, particularly in regions with high mosquito exposure and limited healthcare access. The findings highlight the need for improved diagnostic capacity, targeted public health interventions, and strengthened healthcare infrastructure to support early diagnosis and effective disease management.

## Author Contributions

Surgical and Medical Practices: Ahmed Mohamed Ali, Ahmet Dogan, Fardowso Ali Mohamud, Tigad Abdisad Ali, Suad Abdikarim Isse, and Abdijalil Abdullahi Ali. Concept: Ahmed Mohamed Ali and Ahmet Dogan. Design: Ahmed Mohamed Ali, Ahmet Dogan, and Fardowso Ali Mohamud. Data collection or processing: Ahmed Mohamed Ali, Fardowso Ali Mohamud, Tigad Abdisad Ali, Suad Abdikarim Isse, and Abdijalil Abdullahi Ali. Analysis or interpretation: Ahmed Mohamed Ali and Ahmet Dogan. Literature search: Ahmed Mohamed Ali and Ahmet Dogan. Writing: Ahmed Mohamed Ali and Ahmet Dogan.

## Funding

The authors received no financial support.

## Disclosure

All authors reviewed the results and approved the final version of the article.

## Ethics Statement

Before the commencement of the study, we obtained ethical clearance from the Ethics Committee of the Recep Tayyip Erdogan Training and Research Hospital (approval number: MSTH/13782, 2025). All cases included in the study were informed about the study. All participants gave written informed consent. In addition, permission to publish was obtained from patients for figures containing identifiable images (Figure 3(a)). Participants were informed about the study’s potential risks through a question‐and‐answer session. The form indicating that the cases voluntarily participated in the study was read in detail and signed.

## Consent

Please see the Ethics Statement.

## Conflicts of Interest

The authors declare no conflicts of interest.

## Supporting Information

Additional supporting information can be found online in the Supporting Information section.

## Supporting information


**Supporting Information 1** Supporting file 1: The percentage of regions where the cases included in the research live is shown in a pie chart.

**Supporting Information 2** Supporting file 2: The occupations of the cases included in the research are shown in a bar chart.

## Data Availability

The data are available from the corresponding author upon reasonable request.
