# Supplementary figures and images for: Frequency and Distribution of Lymphatic Filariasis in Somalia: A Single‐Center Experience
Source: J Trop Med. 2026 May 15;2026:7385823. doi: 10.1155/jotm/7385823 (PMC13176848; doi:10.1155/jotm/7385823)

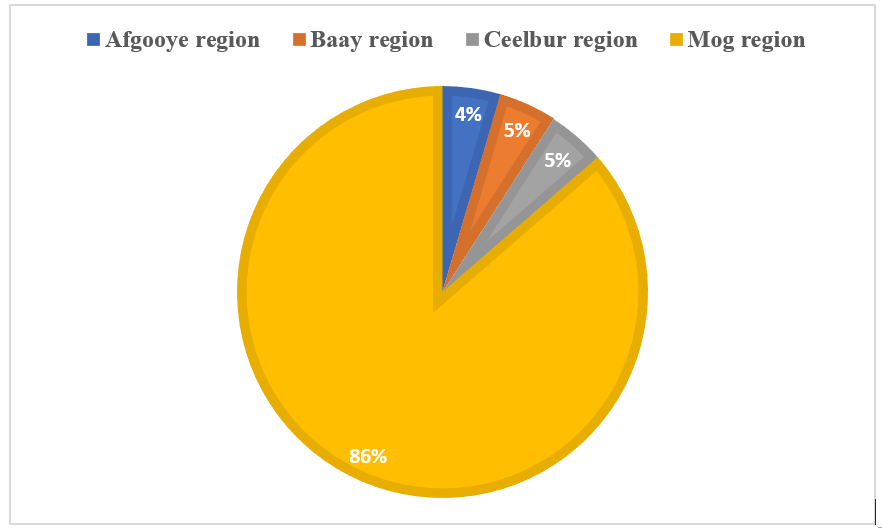


**Graph 1. Regions Where Cases Live**

Supplement: Supplementary file 1 — Supporting Information 1 Supporting file 1: The percentage of regions where the cases included in the research live is shown in a pie chart. [file JOTM-2026-7385823-s002.docx]

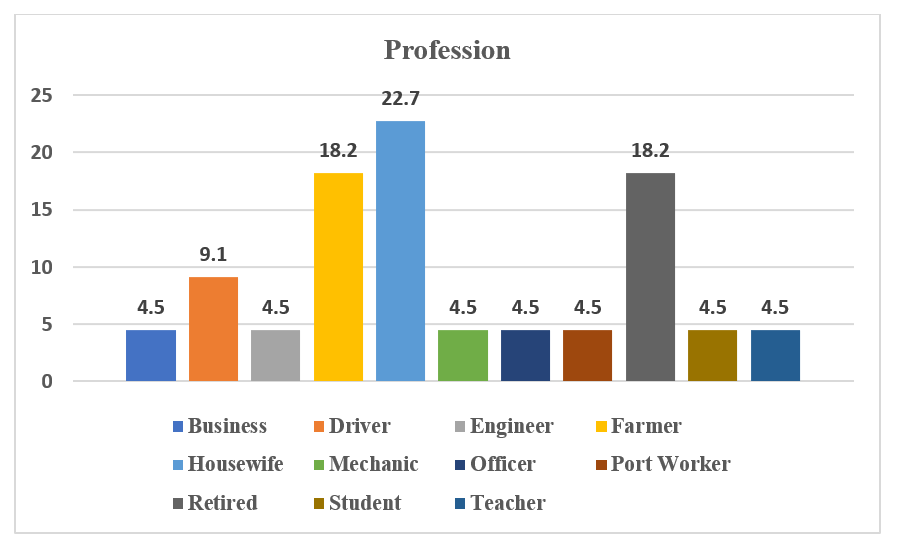


**Graph 2. Occupations (%)**

Supplement: Supplementary file 2 — Supporting Information 2 Supporting file 2: The occupations of the cases included in the research are shown in a bar chart. [file JOTM-2026-7385823-s001.docx]
